# Endoscopic ear surgery – a complement to microscopic ear surgery

**DOI:** 10.1007/s00106-016-0268-x

**Published:** 2016-12-08

**Authors:** S. Preyer

**Affiliations:** Ohrenschwerpunkt Karlsruhe am Diakonissenkrankenhaus, Diakonissenstr. 28, 76199 Karlsruhe, Germany

**Keywords:** Middle ear surgery, Endoscopic surgical procedures, Middle ear, Cholesteatoma, Tympanoplasty, Video-assisted surgery

## Abstract

Wullstein, the founder of modern microscopic ear surgery, already used an oto-endoscope intraoperatively. However, it is only after the recent development of modern video-endoscopy with high-definition, 4K, and 3‑dimensional imaging that endoscopically guided surgery of the middle ear is gaining some importance. Key ventilation routes like the isthmus tympani and the epitympanic diaphragm can be visualized far better using an endoscope than with a microscope. Going through the external meatus, surgery of middle ear pathologies is possible without external incision. This type of primary endoscopic ear surgery has to be distinguished from secondary endoscopic ear surgery, where standard microscopic ear surgery is supplemented by endoscopic surgery. Having to hold the endoscope in one hand, surgery has to be performed single-handedly, which is awkward. In cases of extensive bone removal or excessive bleeding, the view through the endoscope lens is obscured; therefore; the endoscope cannot fully substitute the microscope. It is, however, an interesting adjunct to microscopic ear surgery.

Microscopic surgery is the gold standard for surgeries of the middle ear, mastoid and lateral skull base. In Germany, microscopic ear surgery is performed at a very high level, with very good results with respect to control of pathologies and hearing function. Endoscopic ear surgery is gaining increasing importance internationally as an adjunct to microsurgery and a further development of traditional microscopic ear surgery. However, in Germany, endoscopic ear surgery has not yet gained general acceptance as a routine procedure.

## Background

Although endoscopic ear surgery is still in its infancy, it is gaining increasing attention internationally. The first reason for this increasing interest is the patients’ wish for minimal invasive surgery to avoid an external incision.

The quality of endoscopic images is at least equal to microscopic visualization

Secondly, endoscopic visualization has improved significantly during the past decades due to high-definition (HD) video imaging and wide-field endoscopy, such that today, the quality of endoscopic images is equal or in some aspects maybe even superior to microscopic visualization.

## History

Modern ear surgery is based on use of the operating microscope to visualize the delicate middle ear structures. Our concepts of ear surgery and classification of tympanoplasty were developed in the 20^th^ century. These concepts still determine surgical procedures today [23].

Ohnsorge at the Würzburg ENT clinic was the first to describe intraoperative use of an endoscope [14]. After proposals to use the endoscope for diagnostic purposes [3, 13], Wullstein used an “ototympanoscope” from the company Storz with a diameter of 2.7 mm intraoperatively in 1984. However, the device had to be held in both hands and could therefore only be used for a control look around the corner [30]. Nine years later, Thomassin and McKennan independently proposed a minimal invasive approach and use of the endoscope for second-look surgery after cholesteatoma surgery [10, 31]. Both surgeons introduced the endoscope into the mastoid via a small incision within the course of the retroauricular scar. Thomassin described use of 30 and 70° endoscopes to reduce residual cholesteatoma in the tympanic sinus and the retrotympanum [30]. In the same year, Poe used the endoscopic approach to inspect the round window for perilymphatic fistulas [19] and, in 2000, described endoscopic stapedioplasty for the first time [18]. Tarabichi developed endoscopic ear surgery further and published two case series with 38 and 165 patients in 1997 and 1999, respectively, in whom he performed endoscopic surgery for cholesteatoma and perforations of the tympanic membrane without a microscope [24, 25].

Subsequent publications describe two types of endoscopic ear surgery: a secondary endoscopic approach, i.e. using the endoscope for an additional visual control in microscopic middle ear surgery [20, 31], and primary endoscopic ear surgery, i. e. all steps of surgery are performed endoscopically [4, 6, 11, 24, 29].

## Approach

For endoscopic ear surgery, rigid endoscopes with angles ranging from 0 to 70° and diameters of 2.7 to 4 mm are used. Initial concerns that the heat at the tip of the endoscope might cause tissue damage have since been dispersed. It has been shown that the temperature at the tip of the endoscope is not as great as initially estimated, simply because the endoscope has to be removed from the ear at regular intervals for cleaning purposes. Cleaning the endoscope and applying anti-fog solution allow time to cool down [20].

**Fig. 1:**
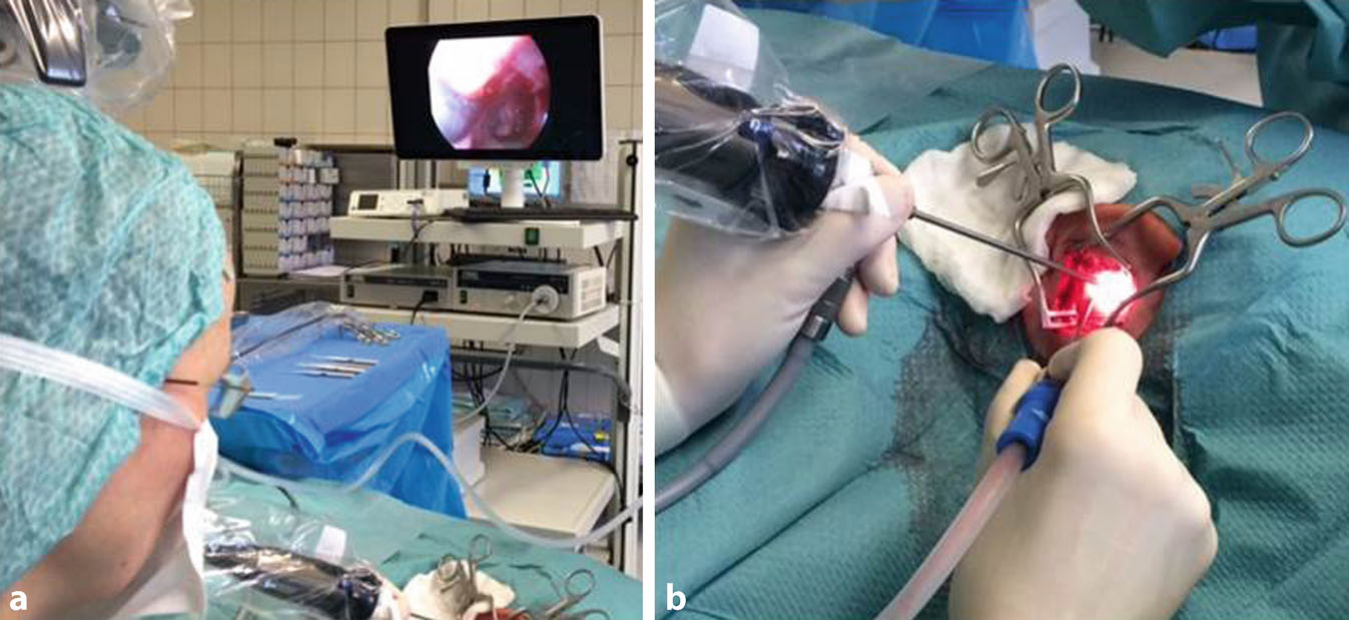
Setup for endoscopic ear surgery. **a** The ready-to-use microscope can be seen in the upper left corner. **b** The left hand holds the endoscope, while the right hand performs the surgery

During endoscopic ear surgery the surgeon holds the endoscope in one hand while working in the ear with the other (Fig. 1). To allow this kind of single-handed surgery, different surgical instrument companies have developed special surgical instruments with suction. Since it is possible to look around corners with the endoscope, curved ear instruments have been developed that enable the surgeon to also work around corners. To avoid time delays in special situations, e. g. heavy bleeding during endoscopic ear surgery, the operating microscope should always be ready for use in the operating room. This allows the surgeon to change to the microscope at any stage of the surgery if necessary.

### Secondary endoscopic ear surgery

Historically, secondary endoscopic ear surgery is the older technique and was developed before primary endoscopic ear surgery with the intention of improving the outcome of cholesteatoma surgery [30]. For viewing around the corner, 30, 45 and 70° endoscopes are used as an adjunct to the microscope to clear cholesteatoma from blind spots, i. e. the epitympanum, retrotympanum, hypotympanum and Eustachian tube orifice [20, 31]. For access to the middle ear or mastoid, a traditional retroauricular or endaural approach is used. Pathologies are removed under the microscope and the posterior wall of the external meatus is either preserved or removed [30].

Use of the endoscope improves visual control around corners and thus results in reduction of the amount of healthy bone to be removed and improved preservation of temporal bone anatomy [1, 11, 24]. This is particularly true for regions like the sinus tympani and hypotympanum, which are hard to reach microscopically even after extensive bone removal [28, 29]. Systematic endoscopic anatomic studies have shown that mastoid cells which extend posterior to the facial nerve and below the jugular bulb cannot be visualized under the microscope and, therefore, residual cholesteatoma is often left behind in these areas [5, 12, 21, 29].

### Primary endoscopic ear surgery

In primary endoscopic ear surgery, the middle ear is approached via the external meatus [25] without an external skin incision [1, 11, 19, 24]. This reduces perioperative soft tissue damage.

The curvature of the external meatus plays no role in endoscopy

Since the endoscope is positioned medial to the natural curvature of the external meatus, the view of the middle ear is much better than when using the microscope. A short tympanomeatal flap is elevated in the osseous portion of the external meatus to access the middle ear [20]. Later, a small incision may be necessary to harvest graft material, such as tragal cartilage or muscle fascia. During surgery, the endoscope remains lateral to the annulus most of the time, as this reduces the risk of damaging the delicate structures in the middle ear. In cases of large tympanic membrane perforations, the anatomy and function of the ossicular chain can be judged through the perforation prior to opening the middle ear. After elevation of the tympanomeatal flap, the oval window can usually be seen without bone removal, which reduces the risk of damage to the chorda tympani. Curved instruments are needed for surgical manipulation of the stapes or the region of the istmus tympani. These instruments are different from the typical straight instruments used for microscopic surgery [18]. Cholesteatoma is removed starting in the middle ear and proceeding into the mastoid, healthy bone only being removed when needed for accessibility. Due to the wide field of vision, less healthy bone needs to be removed in endoscopic than in microscopic surgery. Reconstruction of the ossicular chain and tympanic membrane is performed by traditional techniques; however, this requires some practice, because handling the graft materials with only one hand is more difficult.

### Endoscopic ear anatomy

**Fig. 2:**
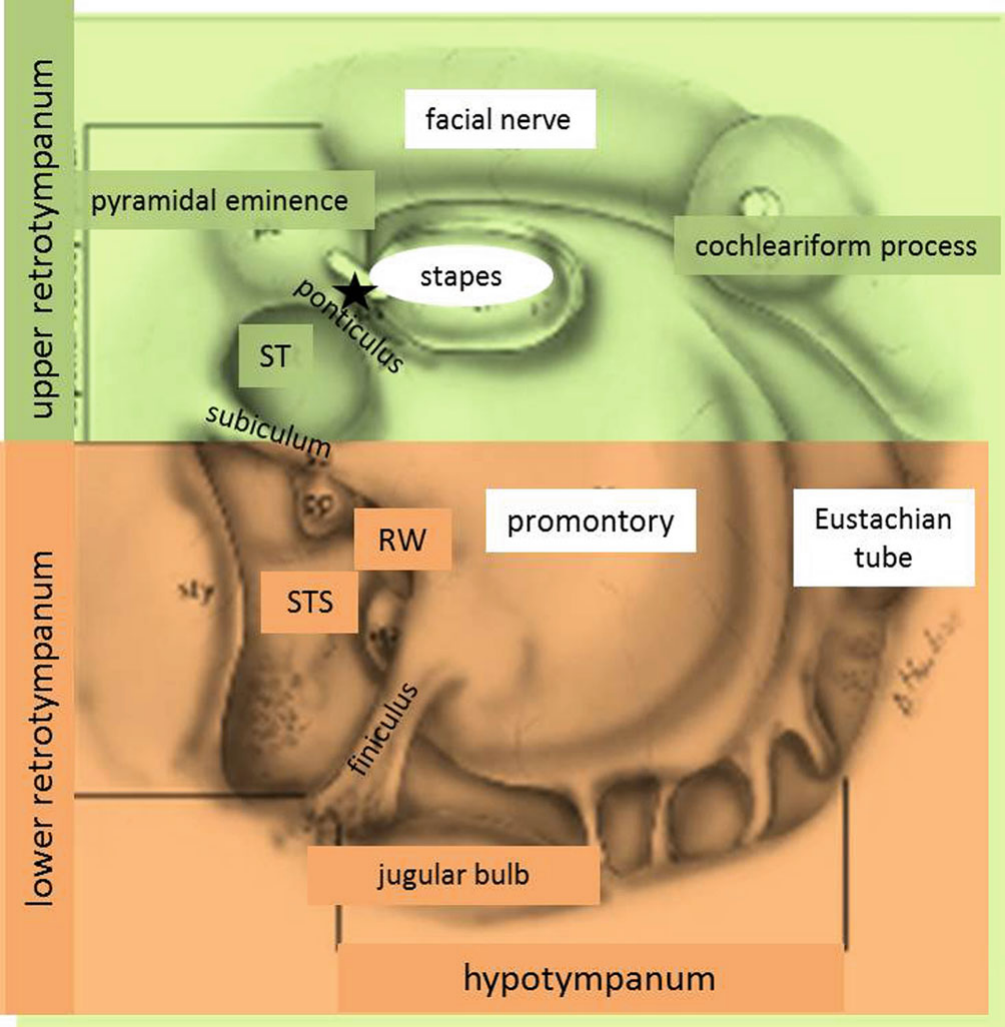
Endoscopic middle ear anatomy. *ST* sinus tympani, *STS* subtympanic sinus, *RW* round window, *star* posterior sinus. (Figure modified from [12] with permission from Elsevier)

The endoscopic transmeatal approach to the middle ear allows the surgeon to see middle ear structures which, in the past, were hidden from view using the microscope. An adequate surgical anatomy had to be developed to describe endoscopic surgical steps in the middle ear. It is possible that the changed view of middle ear anatomy will improve cholesteatoma surgery, as cholesteatoma growth normally follows given anatomic routes and precise knowledge of osseous anatomy should therefore improve its removal. Since 2009, Daniele Marchioni and coworkers have published several papers on endoscopic middle ear anatomy and suggest a nomenclature and new classification of structures according to practical aspects ([4, 7–9; Fig. 2). These authors define an upper and lower retrotympanum, which are separated from the hypotympanum by the finiculus (earlier name: sustentaculum promontorii) [5, 9]. If the finiculus is bridge shaped, cholesteatoma can grow from the round window niche along infracochlear cells towards the petrous apex [9]. Of the middle ears examined in a clinical study of children undergoing middle ear surgery, 90% were found to have a bridge-shaped finiculus, whereas this was the case in only 60% of adult ears [9]. The upper and lower retrotympanum are divided by the subiculum. Marchioni describes recurrent cholesteatoma as originating most often from the subtympanic sinus between the subiculum and finiculus [5], particularly when it extends far posterior to the mastoid segment of the facial nerve or even posterior to it.

**Fig. 3:**
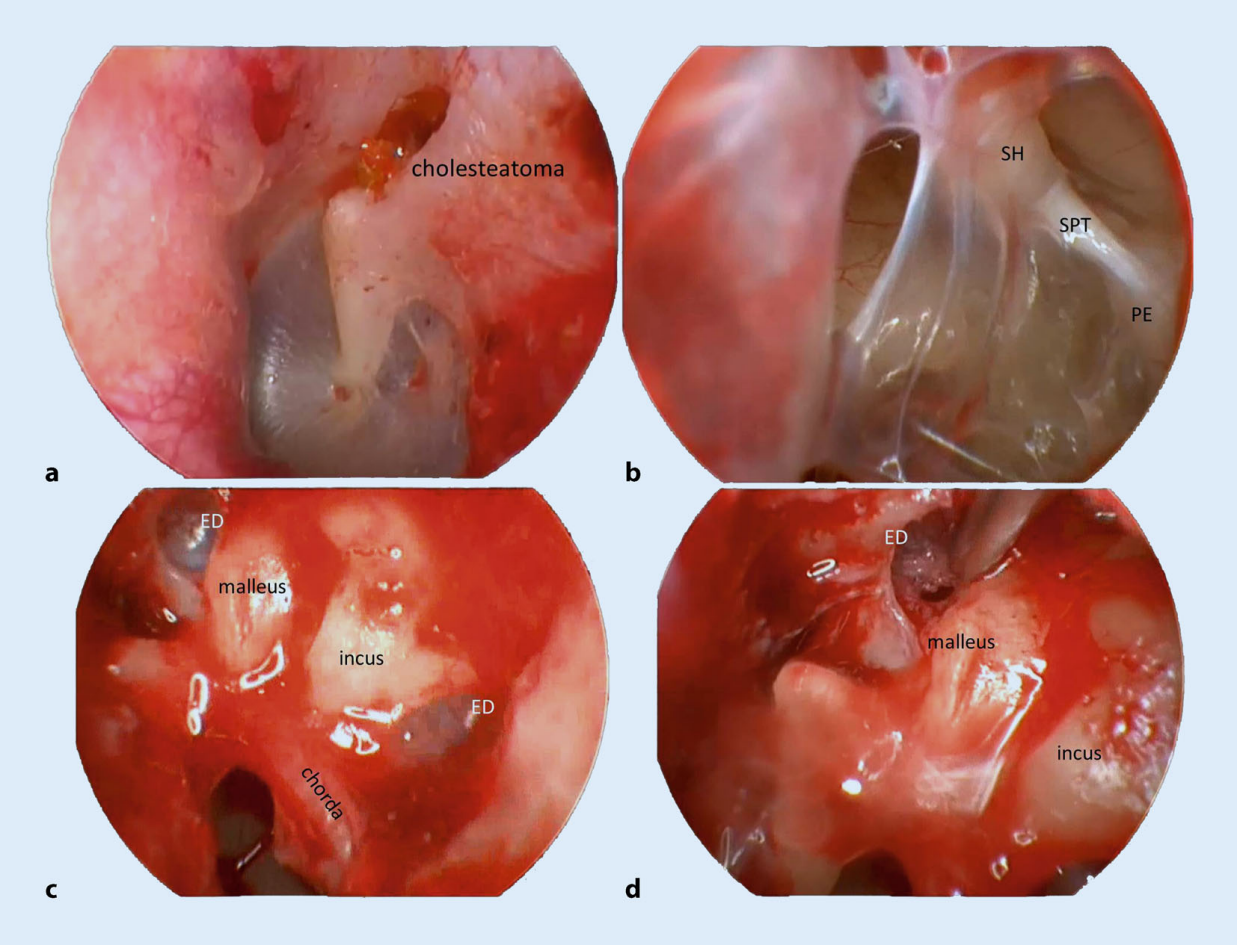
Endoscopic middle ear surgery in a patient with epitympanic dysventilation. **a** Epitympanic cholesteatoma with an otherwise normal tympanic membrane. **b** Mucosal folds obstructing the isthmus tympani. **c** Complete epitympanic diaphragm. **d** Dissection of the anterior malleolar fold to open up the epitympanic space for ventilation via the supratubal recess. *SPT* stapedius tendon, *SH* stapes head, *ED* epitympanic diaphragm, *PE* pyramidal eminence

Endoscopically, the anterior epitympanum and the supratubal recess can always be visualized and, therefore, the tensor fold can be accessed for surgical manipulations [4, 8, 17]. In 1946, Chatellier and Lemoine described and named the epitympanic diaphragm, consisting of the anterior and lateral malleolar ligaments, as well as the posterior and lateral incudal ligaments which separate the epi- and mesotympanum ([2, 17]; Fig. 3). Under normal conditions, the epitympanum is aerated exclusively via the tympanic isthmus [15, 21], which is limited by the tensor tympani tendon anteriorly and the medial part of the posterior incudal ligament posteriorly. Endoscopically, it is possible to judge the patency of the tympanic isthmus and to remove mucosal folds or granulation tissue obstructing it. Prussak’s space is ventilated via the pocket of von Tröltsch posteriorly and is independent of the epitympanic space—both anatomically and with respect to ventilation [16].

## Epitympanic cholesteatoma

### Pathophysiology from an endoscopic point of view

Based on endoscopic observations of middle ear ventilation routes, it has been hypothesized that selective dysventilation of the epitympanum may be a mechanism for development of epitympanic cholesteatoma [21]: the typical and not rare finding of an epitympanic cholesteatoma with a normal tympanic membrane and positive Valsalva manoeuvre is often associated with a retracted malleus and a reduced distance between the handle of the malleus and the long process of the incus, or with a blocked tympanic isthmus due to congenital or acquired mucosal folds or granulation tissue ([6]; Fig. 3). According to this new hypothesis, retraction of the pars tensa of the tympanic membrane and atelectasis of Prussak’s space are two distinct independent phenomena leading to cholesteatoma [16].

### New therapy possibilities

Performing endoscopic surgery for epitympanic cholesteatoma allows the surgeon to check the epitympanic diaphragm and the tensor fold. A new ventilation pathway via the supratubal recess can be created by dissecting a complete diaphragm and tensor fold ([4, 6]; Fig. 3).

It has been proposed that this surgical manoeuvre might improve ventilation of the epitympanic space and help in preventing recurrent cholesteatoma. Furthermore, ventilation of the epitympanic space may be improved by the removal of mucosal folds and granulation tissue from the tympanic isthmus [4]. These surgical manoeuvres around the corner can only be performed endoscopically. Thus, endoscopic ear surgery opens a new avenue of functional surgery, with the goal of also improving ventilation [8].

Extensive bone removal is necessary to microscopically remove cholesteatoma deep in the hypotympanum or far posterior in a deep sinus tympani extending far beyond the facial nerve. By contrast, more healthy bone can be preserved endoscopically and cholesteatoma is removed under good visual control [5, 9].

Improved ventilation of the epitympanic space can prevent recurrent cholesteatoma

In 2013, Marchioni et al. proposed a transmeatal, transtympanal endoscopic approach to the internal auditory meatus as an alternative route to the traditional translabyrinthine approach [7]. In their anatomic studies, the internal auditory meatus and the complete course of the facial nerve could be exposed transmeatally without an external skin incision.

## Discussion

The basic principles of endoscopic ear surgery are similar to those of microscopic ear surgery, but the combination of both extends surgical possibilities.

Based on her team’s own experience with 37 endoscopic ear surgeries—27 primary and 10 secondary cases—the author believes that use of the endoscope improves preservation of the ossicular chain. The risk for damage of the chorda tympani is reduced because the oval and round windows can be visualized endoscopically without removal of the scutum. In cases of second-look surgery, it was possible to look around the corner and preserve previous reconstructions of the ossicular chain or cartilage grafts. Removal of cholesteatoma in the retrotympanum was more reliable with endoscopic visualization and in one case with recurrent cholesteatoma in the extensive hypotympanum, with exposed internal carotid artery and a very deep posterior retrotympanum, removal was only possible endoscopically.

The author’s team has performed transmeatal endoscopic surgery in four children aged 4–7 years with congenital cholesteatoma. The ossicular chains could be preserved in four cases; in one case, the stapes and incus had already been destroyed by the cholesteatoma. Heavy bleeding and extension of disease posterior to the semicircular canal into the mastoid limit use of the endoscope, such that the microscope was used in these cases. Training of young surgeons is easier in endoscopic ear surgery because trainer and trainee have the same view of the surgical field. It was also found that the whole team working in the operating room was more involved in cases where surgery was performed endoscopically, as everybody can follow all surgical steps on the screen “through the surgeon’s eyes”.

Endoscopic surgery of cholesteatoma improves preservation of the ossicular chain

In spite of these advantages, endoscopic ear surgery is spreading only slowly in Germany. Presumably this is because new surgical concepts have to be developed in conjunction with a new approach to the middle ear and also due to the limitations of one-handed surgery. Since the new concepts are scarce and there are only a limited number of valid clinical studies showing the benefit of endoscopic ear surgery **(**Table 1 **)**, most experienced ear surgeons are cautious of adopting the new technique into clinical routine at present.

**Table 1:** Clinical studies on endoscopic ear surgery

Publications in chronological order	Number of cases	Aim of study	Results	Type of study
McKennan K.X. 1993 [10]	12 patients	Recurrent cholesteatoma Endoscopic second-look ear surgery: retroauricular incision after microscopic canal wall up surgery	No postoperative loss of sensitivity Less morbidity No complications	Case series
Thomassin J.M. et al. 1993 [31]	80 patients	Cholesteatoma Comparison of A: 44 microscopic canal wall up procedures with B: 36 combined microendoscopic canal wall up procedures	Recurrent cholesteatoma at second-look after 12–18 months: Group A: 47.7% Group B: 5.5%	Retrospective study on two consecutive case series
Tarabichi M. 1997 [24]	36 patients	Cholesteatoma	Clinical follow-up: 1 year postoperatively: 29/30 patients no cholesteatoma 2 years postoperatively 10/13 patients no cholesteatoma Second-look after 2 years: 4/6 patients no cholesteatoma	Case series
Tarabichi M. 1999 [25]	165 patients	96 tympanoplasties 13 stapesplasties 56 cholesteatomas	88% tympanic membrane perforation closed, “Results comparable to literature and own historical group of patients”	Retrospective study
Poe D.S. 2000 [18]	34 patients	Laser-assisted stapedioplasty: 11 stapes mobilisation 17 endoscopic stapedotomy 6 microscopic stapedotomy	Hearing at 0.5,1,2,3 kHz after 6 months as in historical group of patients No complications Improved visualization of anterior crus and anterior third of footplate with endoscope	Prospective study
Tarabichi M. 2000 [26]	69 ears	Cholesteatoma	In 3 cases, changeover to microscope with retroauricular incision Mean follow-up 41 months 6 recurrent cholesteatoma	Retrospective study
Tarabichi M. 2004 [27]	73 ears	Epitympanic cholesteatoma	Mean follow-up 43 months: 5 recurrent cholesteatoma 8 recurrent perforations or chain defects No bone conduction loss or facial nerve injury	Case series
Migirov L. et al. 2011 [11]	20 patients	Cholesteatoma	Clinical follow-up after 1 year: 18 patients no cholesteatoma 12 patients not yet re-examined	Retrospective study
Sarkar S. et al. 2013 [22]	32 patients	Stapedotomy: 30 endoscopic 2 microscopic due to gusher	Air–bone gap 0.5, 1, 2, 4 kHz Preoperative 41.5 + 5.2 dB 3 months postoperatively 10.1 + 3.6 dB Bone conduction change 0.1 + 0.7 dB	Case series

Intraoperative use of endoscopes in middle ear surgery has already opened up new perspectives in ear surgery, in spite of the slow spread of the method. In the future, endoscopic ear surgery will become an indispensable adjunct to microscopic ear surgery.

## Conclusion

- Transmeatal endoscopic ear surgery is a promising new technique.
- The transmeatal approach reduces perioperative soft tissue damage.
- Wide-field 0 and 30 or 70° endoscopes allow visualization of hidden anatomic spaces and working around corners, i. e. epitympanum, hypotympanum and retrotympanum, for safe removal of cholesteatoma.
- Visual control of ventilation pathways, i. e. tensor fold and isthmus tympani, allow surgical manipulations improving air passage to the epitympanic space.
- Endoscopic anatomic studies indicate that selective dysventilation of the epitympanum may be a mechanism inducing epitympanic cholesteatoma in patients with normal Eustachian tube function.
- In the future, the new endoscopic approach to middle ear pathologies will also change the microscopic approach to the ear.
